# Extreme diversity and multiple SCC*mec* elements in coagulase-negative Staphylococcus found in the Clinic and Community in Beijing, China

**DOI:** 10.1186/s12941-017-0231-z

**Published:** 2017-08-22

**Authors:** Xiao-Ping Chen, Wen-Ge Li, Hao Zheng, Hai-Yan Du, Li Zhang, Lei Zhang, Jie Che, Yuan Wu, Shu-Mei Liu, Jin-Xing Lu

**Affiliations:** 1 State Key Laboratory for Infectious Disease Prevention and Control, Collaborative Innovation Center for Diagnosis and Treatment of Infectious Diseases, National Institute for Communicable Disease Control and Prevention, Chinese Center for Disease Control and Prevention, Beijing, 102206 China; 2 Microbiology Laboratory, Fu Xing Hospital, Capital Medical University, Beijing, 100038 China; 3 FuXingMenWai Road 20, XiCheng, Beijing, 100038 China; 4 Changbai Road 155, ChangPing, Beijing, 102206 China

**Keywords:** Coagulase-negative staphylococci, Multiple SCC*mec*, Bacterial cell wall integrity

## Abstract

**Background:**

Coagulase-negative staphylococci (CoNS) are recognized as a large reservoir of staphylococcal cassette chromosome *mec* (SCC*mec*) harboured by *Staphylococcus aureus*. However, data of SCC*mec* in CoNS are relatively absent particularly in China.

**Methods:**

Seventy-eight CoNS clinical and 47 community isolates were collected in Beijing. PCR was performed to classify SCC*mec* types. Under oxacillin treatment, quantitative real-time reverse transcription PCR (qRT-PCR) was performed to compare *mecA* mRNA levels and mRNA half-life between isolates with single SCC*mec* element and those with multiple one. Their growth curves were analysed. Their bacterial cell wall integrity was also compared by performing a Gram stain. All *ccr* complex segments were sequenced and obtained *ccr* segments were analysed by phylogenetic analyses.

**Results:**

All 78 clinical isolates had *mecA* segments compared with 38% in community isolates (total 47). Only 29% clinical isolates and 33% community isolates (among *mecA* positive isolates) harboured a single previously identified SCC*mec* type; notably, 17% clinical isolates and 28% community isolates had multiple SCC*mec* types. Further studies indicated that isolates with multiple SCC*mec* elements had more stable *mecA* mRNA expression compared with isolates with single SCC*mec* elements. CoNS with multiple SCC*mec* elements demonstrated superior cell wall integrity. Interestingly, phylogenetic analyses of obtained 70 *ccr* segments indicated that horizontal gene transfer of the *ccr* complex might exist among various species of clinical CoNS, community CoNS and *S. aureus*.

**Conclusions:**

CoNS recovered from patients carried extremely diverse but distinctive SCC*mec* elements compared with isolates from the community. More attention should be given to CoNS with multiple SCC*mec* not only because they had superior cell wall integrity, but also because CoNS and *S. aureus* might acquire *multiple SCCmec* through the *ccr* complex.

**Electronic supplementary material:**

The online version of this article (doi:10.1186/s12941-017-0231-z) contains supplementary material, which is available to authorized users.

## Background

Coagulase-negative staphylococcus (CoNS) is a part of the commensal bacterial microflora of healthy people. However, with the development of interventional therapy and the increasing number of immunocompromised patients, these bacteria are becoming the most important causes of nosocomial infections [1, 2]. CoNS bloodstream infections have been estimated in as many as 250,000 cases annually in the US. The mortality rate of these infections is 1–25%, representing a great burden to the public health system [3]. The most common CoNS in nosocomial infection are *Staphylococcus epidermidis*, followed by *Staphylococcus haemolyticus*, *Staphylococcus hominis* and *Staphylococcus capitis* [4]. Another important reason for the increasing concern for CoNS is the fact that they also harbour SCC*mec* elements, which are found in methicillin-resistant *S. aureus* (MRSA). SCC*mec* elements harbour *mec* genes (*mecA/mecC*), providing resistance to methicillin and nearly all other beta-lactam antibiotics [5].

In general, SCC*mec* has two essential components, i.e., the *mec* gene complex and the cassette chromosome recombinase (*ccr*) gene complex. The *mec* gene complex consists of *mecA/mecC*, regulatory genes and associated insertion sequences and has been classified into five main classes, i.e., class A, class B, class C1, class C2, class D, which has been observed only in *Staphylococcus caprae*, and newly found class E [6, 7]. Encoding recombinases mediating integration and excision of SCC*mec* into and from the chromosome, *ccr* genes (*ccrC* or the pair of *ccrA* and *ccrB*) play an important role in the transfer of SCC*mec* elements [8]. The *ccr* gene(s) and surrounding genes form the *ccr* gene complex. At present, two distinct *ccr* gene complexes have been reported based on the composition of *ccr* genes, one carrying two adjacent *ccr* genes, *ccrA* and *ccrB*, and the second carrying *ccrC*. The *ccrA* and *ccrB* genes identified in *S. aureus* strains are categorized into four and five allotypes respectively, resulting in six *ccr* gene complex types, designated as type 1 (*ccrA1B1*), type 2 (*ccrA2B2*), type 3 (*ccrA3B3*), type 4 (*ccrA4B4*), type 7 (*ccrA1B6*) and type 8 (*ccrA1B3*). In contrast, all identified *ccrC* variants to date show high nucleotide similarity and are designed to only one allotype, *ccrC1*, constituting type 5 of *ccr* gene complex [7, 9]. Because of the high diversity of *ccr* gene complex and *mec* gene complex, an extensive genetic diversity of SCC*mec* elements has been revealed in *S. aureus* and a total of twelve types of SCC*mec* have been assigned for *S. aureus* based on the classes of the *mec* gene complex and *ccr* gene complex [9].

Previous studies have found that specific SCC*mec* elements, or components, exist in particular CoNS. For example, type IV was preferentially associated with *S. epidermidis* and type V was prevalently found in *S. haemolyticus* in the hospital [10, 11]. However, in recent years, more diverse SCC*mec* elements including non-*mecA*-encoding cassettes had been revealed from CoNS, and many SCC*mec* elements in methicillin-resistant CoNS (MR-CoNS) could not be typed using currently available schemes applied to MRSA. Moreover *mecA* gene has been found more widely distributed among CoNS than among *S. aureus* indicating a potential reservoir for the transfer of SCC*mec* elements to *S. aureus* [10, 12, 13]. However, only a small number of SCC*mec* elements of CoNS have been characterized in China [14]. In addition, the precise role of SCC*mec* elements of CoNS in the emergence and evolution of MRSA remains obscure, which requires characterization of additional SCC*mec* elements. Furthermore, multiple SCC*mec* have been found in CoNS recovered from patients in several studies [14, 15]. To obtain information on the SCC*mec* of local CoNS in Beijing and to reveal the function of multiple SCC*mec* in CoNS, clinical and community isolates were investigated and the features (including *mecA* mRNA quantity, mRNA half life, growth curve, bacterial cell wall integrity) of CoNS with multiple SCC*mec* elements were compared with those harbouring a single SCC*mec* element.

## Methods

### Sample collection and bacterial isolation

Only one isolate from each subject was collected and further analysed in the study. The demographic, hospital, and microbiological data were anonymously collected. Clinical CoNS isolates were collected from two hospitals in Beijing from July 2013 to December 2015. These CoNS strains were isolated from the blood of inpatients. The ages ranged from 34 to 98 years (mean ± SD, 50 ± 16). These patients were hospitalized for more than 48 h and were suspected of having a blood bacterial infection. Collected blood samples were inoculated into aerobic BacT/Alert FAN blood culture bottles and incubated in the BACT/Alert machine (bioMérieux, Marcy l’Etoile, France) for up to 5 days. Positive culture samples were directly inoculated onto Mueller–Hinton Broth (MH broth, Oxoid LID, Basingstoke, Hampshire, England) supplemented with 2% NaCl and incubated aerobically at 37 °C for 72 h. Species identification were determined using the Vitek II (bioMerieux, Durham, NC, USA) automated microbiology system and further confirmed by partially sequencing 16S rRNA genes amplified with primers 5F and 1194R and *rpoB* genes with primers 2491F and 3241R [13].

Community CoNS isolates were collected from healthy subjects (aged from 20 to 48) in two communities in Beijing in June 2016. Three groups of subjects, including office workers, construction workers and soldiers, were recruited. Samples were collected from the forehead and elbow with cotton swabs wetted with sterilized PBS. The swabs were placed into Mueller–Hinton Broth (MH broth, Oxoid LID, England) supplemented with 2% NaCl and incubated aerobically at 37 °C for 72 h. Ten microliters of culture suspected of bacteria growth were inoculated onto Brain Heart Infusion (BHI, Oxoid LID, England) agar and suggestive colonies with white color and smooth edge were subjected to screening tests with partial sequencing of 16S rRNA and *rpoB* genes as described above.

### Detection of *mec* gene and SCC*mec* typing

The existence of the *mecA* gene was identified using primers met1/met2 and *mecC* gene with primers mecCF/mecCR [5, 16]. For *mecA* positive isolates, SCC*mec* typing was defined by the combination of *ccr* type and *mec* class, which were obtained using PCR [6, 9]. The *mec* class was assigned with five primers to identify the gene lineages of *mecA*–*mecI* (class A *mec* with primer mA7/mI6), *mecA*-IS1272 (class B *mec* with primer mA7/IS7), *mecA*-IS431 (class C *mec* with primer mA7/IS2) and *mecA*-IS431L (class C1 *mec* with primer mA7/IS2L). To further discriminate class C1 or C2 *mec* complexes, sequences between IS431 and *mecA* were examined using PCR with primer (IS431-F2 or IS431-R1) located in either direction of IS431 paired with primer mecA-R2 in *mecA* [14]. Five *ccr* gene complexes were identified with eight primers: four primers consisting of a common reverse primer (common to *ccrB1*-*3*, i.e., primer BC) and three forward primers specific for *ccrA1*, *ccrA2*, and *ccrA3* to confirm *ccr1*–*3* based on differences in *ccrA* genes; two primers to identify *ccr4*; and two primers to identify *ccrC* [6, 11].

For *mecA* negative isolates, *ccr* complexes were also analysed with primers as described above. The primers and lengths of amplicons used to identify the *mec* gene, *mecA* classes and *ccr* complexes are listed in Additional file 1: Table S1. PCR products of *ccr* complexes from all isolates were sent to Sangon Biotechnology Company (Sangon Biotech, Shanghai, China) for sequencing. The results were blasted with sequences in GenBank, and *ccr* genes with nucleotide identities more than 85% were designed to the same allotype [9].

Those with two *mecA* classes were designated CoNS with multiple SCC*mec* elements; those with one class of *mecA* complex and one *ccr* complex detected were classified into CoNS with a single SCC*mec* element. All strains with multiple SCC*mec* elements and those with single elements were further characterized.

### Determination of minimal inhibitory concentrations (MIC) to oxacillin

For MIC determination, a broth microdilution broth susceptibility assay was performed according to CLSI guidelines [17]. The oxacillin (oxacillin sodium monohydrate, Sigma-Aldrich, St. Louis, MO, USA) concentration ranged from 256 to 0.125 μg/ml. The plates were incubated under normal atmospheric conditions for 24 h at 37 °C. The presence of a white pellet on the bottom of the tube indicated bacterial growth. The MIC value was identified by the lowest concentration of oxacillin at which no visible growth could be observed.

### Growth curves of CoNS under oxacillin treatment

Samples of bacterial culture were prepared as follows: a single colony of the strain was cultured with MH broth overnight at 37 °C. Bacterial suspensions were diluted with MH broth to 0.5 McFarland standards and added to oxacillin (final concentration 2 μg/ml) and cultured at 37 °C. Two millilitres of culture was removed at the indicated time point. The growth curves were measured by plate counts on MHI agar (Oxoid LID, Basingstoke, Hampshire, England). The experiments were repeated three times, and the results were reported as an average of the replicate samples.

### Quantification of *mecA* mRNA in CoNS under oxacillin treatment

Samples of bacterial culture were prepared as described above. Total RNA was extracted using the OMEGA bacterial RNA kit (OMEGA Biotech, Doraville, GA, USA) and eluted into 50 μl ddH_2_O. Three microliters of RNA was reverse transcribed into cDNA using the TransScript TM Two-Step RT-PCR Super Mix (TransGene Biotech, Beijing, China) in a total volume of 20 μl. Using standard PCR and real-time PCR, 2 μl of cDNA was used to evaluate and represent the quantity of total *mecA* mRNA in each 2 ml sample because it held the same proportion of total RNA in each sample.

For standard PCR, 2 μl of cDNA was amplified via PCR with TransScript 2× PCR Super Mix (Transgene Biotech, Beijing, China) in a total volume of 30 μl. The primer pairs (mecAF/mecAR) are shown in Additional file 1: Table S1 [18].

For qRT-PCR analyses, real-time PCR amplification was performed in a total volume of 25 μl containing 12.5 μl of 2× SYBR Fast qPCR Mix (TAKARA, Dalian, China), 1.0 μl primer and 2 μl template cDNA. The specific primers (mecA-1501F, mecA-1598R) used for the detection of *mecA* gene are listed in Additional file 1: Table S1 [19]. Data were presented as the relative copies of *mecA* mRNA levels compared with that of untreated CoNS with a single SCC*mec* element.

### *mec*A mRNA half-life identification

Samples of bacterial culture were prepared as described above except with oxacillin cultured at 37 °C for 3 h. Transcriptional arrest was induced with actinomycin D as references except that the dosage of actinomycin D was modified to 2 μg/ml according to a preliminary experiment [20]. Two millilitres of culture was removed at the indicated time point. Total RNA was extracted and subjected to *mecA* mRNA analyses as described above.

### Bacterial cell wall integrity assays

Samples of bacterial culture were prepared as described above except with 8 μg/ml oxacillin treatment. Samples were collected for Gram stain at 0, 1, and 3 h to visualize the bacterial cell wall integrity under a microscope.

### Phylogenetic analyses of *ccr*

The reference sequences of the *ccr* complex in GenBank (Additional file 1: Table S2) and those derived here (Additional file 1: Tables S3, S4) were used to construct a phylogenetic tree. Using MEGA version 5.0, neighbour-joining trees were constructed with the maximum composite likelihood model assuming rate uniformity and pattern homogeneity.

### Statistical analysis

Statistical analysis and graphic presentations were performed with Microsoft Excel XP software. The results are expressed as the average of three assays. A *P* value of 0.05 (Student’s t test) was considered significant.

## Results

### Sample collection

In this study, a total of 78 clinical CoNS isolates and 47 community CoNS isolates were recovered and identified to species.

### Identification of CoNS

CoNS obtained from the clinic were classified into 4 different Staphylococcal species. These included *S. epidermidis* (n = 30), *S. hominis* (n = 20), *S. capitis* (n = 15) and *S. haemolyticus* (n = 13). However, most of the CoNS recovered from community were *S. epidermidis* (n = 40), and the other CoNS were few, including *S. hominis* (n = 5) and *S. haemolyticus* (n = 2).

### Extremely diverse SCC*mec* types and multiple SCC*mec* elements

No *mecC* gene was detected in all these isolates. Not surprisingly, *mecA* was detected in 100% (78/78) clinic isolates compared to 38% (18/47) of the community isolates. The SCC*mec* typing results of these isolates are summarized in Table 1. Interestingly, only a small portion of CoNS were assigned as harbouring a single previously identified SCC*mec* type in both the clinical and community strains (23/78, 29% and 6/18, 33%, respectively). Those identified in clinical isolates included SCC*mec* type III (n = 7), type V (n = 7), type IV (n = 3), type VIII (n = 3), type II (n = 2), and type IX (n = 1). For community strains, only six strains of *S. epidermidis* were confirmed to harbour single SCC*mec* type II. Moreover, 11% (9/78) of clinical CoNS and 5% (1/18) of community isolates recovered carried a new single SCC*mec* type. Strains with an previously identified single SCC*mec* type or new single SCC*mec* type were assigned as CoNS with a single SCC*mec* element.

**Table 1:** Detection of *mecA* segments and SCC*mec* typing results of CoNS isolates

Character of SCC*mec*	Source	Species	*mecA* PCR	*mec*A class	*ccr* type	SCC*mec* type	No. of isolate	Example isolate
Previously identified single SCC*mec* type	Clinic	*S. homi*	+	A	4	VIII	3	H80, H86
			+	A	2	II	1	H59
		*S. epid*	+	A	3	III	4	H8
			+	B	2	IV	3	H67, H11
			+	C2	5	V	2	H81
			+	A	2	II	1	
		*S. haem*	+	C2	1	IX	1	
		*S. capi*	+	A	3	III	3	H4
			+	C2	5	V	5	H7, H26, H54, H60, H85
						*Total number*	*23*	
	Community	*S. epid*	+	A	2	II	6	
						*Total number*	*6*	
New identified single SCC*mec* type	Clinic	*S. homi*	+	A	1	New	3	H29, H34
		*S. haem*	+	C1	4	New	5	H2
			+	C1	2	New	1	
						*Total number*	*9*	
	Community	*S. haem*	+	C1	2	New	1	CJ31-1
						*Total number*	*1*	
New type with a single *mecA* class and multi *ccr* complexes	Clinic	*S. homi*	+	A	1, 4	New	2	H1
			+	A	1, 2	New	1	H73
			+	A	2, 4	New	2	
			+	A	3, 4	New	1	HA1
			+	A	1, 4, 5	New	2	H6
			+	A	1, 3, 4, 5	New	1	
		*S. epid*	+	A	1, 3	New	5	H22
			+	A	1, 3, 4	New	2	H21
			+	A	3, 4, 5	New	1	H92
			+	C2	4, 5	New	1	H76
			+	C2	1, 2, 5	New	1	
			+	B	2, 4	New	3	H24, H77
		*S. haem*	+	A	1, 2, 4	New	1	
			+	C2	2, 4	New	1	H28
			+	C2	4, 5	New	1	H68
			+	C2	1, 4	New	2	H15, H36
		*S. capit*	+	A	3, 4	New	1	H23
			+	A	1, 2, 4, 5	New	1	H37
			+	A	1, 2, 3, 5	New	1	H83
			+	A	1, 2, 3, 4, 5	New	1	H72
			+	C2	2, 4, 5	New	1	H78
			+	C2	1, 2, 4, 5	New	1	
						*Total number*	*33*	
New type with a single *mecA* class and multi *ccr* complexes	Community	*S. epid*	+	A	2, 4	New	1	
			+	A	2, 4, 5	New	1	
			+	B	1, 2	New	3	
			+	B	2, 4	New	1	
						*Total number*	*6*	
New type with multi SCC*mec*	Clinic	*S. homi*	+	A, C2	1, 4, 5	New	1	
			+	A, C2	1, 2, 4, 5	New	2	H33-2
			+	A, C2	1, 2, 3, 4, 5	New	1	H62
		*S.epid*	+	A, B	2, 4, 5	New	1	
			+	A, B	2, 3, 5	New	1	
			+	A, B	2, 3, 4, 5	New	1	H30
			+	A, B	1, 2, 3, 4, 5	New	1	
			+	A, C2	1, 5	New	1	
			+	A, C2	2, 4, 5	New	1	H87
			+	A, C2	1, 2, 3, 4, 5	New	1	H57
		*S. haem*	+	A, B	1, 2, 3, 4	New	1	HA11
		*S.capit*	**+**	A, C2	1, 2, 4, 5	New	1	H14
						*Total number*	*13*	
	Community	*S. homi*	+	A, B	2	New	1	
			+	A, B	1	New	1	CV34
			+	A, B	2, 4	New	1	C3-1
		*S. epid*	+	A, B	1, 2	New	1	
			+	A, C2	2, 5	New	1	
						*Total number*	*5*	
Isolates without *mecA* detected	Community	*S. homi*	−	−	2	N	2	C13-2
		*S. epid*	−	−	2	N	15	C5-1, CJ29
			−	−	1, 2	N	1	
			−	−	2, 5	N	2	
			−	−	5	N	2	CJ28-3
			−	−	2, 4	N	1	CV33-1
			−	−	–	N	5	
		*S. haem*	−	−	2, 4	N	1	
						*Total number*	*29*	

*S. homi, S. hominis; S. epid, S. epidermidis; S. haem, S. haemolyticus; S.capit, S. capitis,* −, *mecA* negative; N, not available

Interestingly, 54% (42/78) of clinical isolates and 39% (7/18) of community isolates (including new single SCC*mec* types, new types with a single *mecA* class and multiple *ccr* complexes) could not be classified into any SCC*mec* type, most of them carried more than one *ccr* complex. Surprisingly, 17% (13/78) of the clinical isolates and 28% (5/18) of the community isolates had two classes of SCC*mec* types (assigned as CoNS with multiple SCC*mec* elements), particularly some clinical isolates that harboured five *ccr* complexes (i.e., *ccrA1B1*, *ccrA2B2*, *ccrA3B3*, *ccrA4B4* and *ccrC*).

Strains (both clinical and community isolates) that did not fit into the SCC*mec* typing criteria, including those harbouring multiple SCC*mec* elements, were designated new SCC*mec* types (total n = 67), which accounted for approximately 70% of the total CoNS with *mecA* segments determined. It was unexpected that among the 29 community strains without a *mecA* or *mecC* segment detected, 24 (83%) contained a *ccr* complex. Furthermore, multiple *ccr* complexes were detected in 5 (21%) strains.

### More stable *mecA* mRNA transcription in CoNS with multiple SCC*mec* elements compared to single elements

**Fig. 1:**
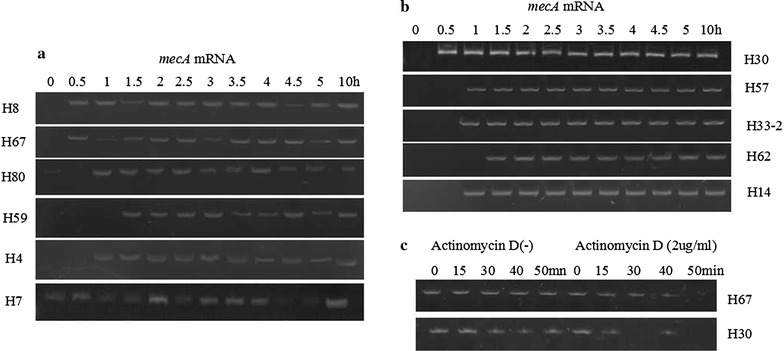
mRNA expression analyses of the amplified fragments of *mecA* in CoNS with single SCC*mec* elements (**a**) and those with multiple SCC*mec* elements (**b**). Two-millilitres culture of CoNS treated with oxacillin (2 μg/ml) for 10 h were used for *mecA* mRNA transcription analyses by reverse transcription PCR (RT-PCR); after treatment with oxacillin (2 μg/ml) and actinomycin D (2 μg/ml), *mecA* mRNA half-lives were determined by RT-PCR (**c**). mRNA expression levels were described in terms of intensity using Quantity One Imager. Data are shown as the image of three independent experiments with similar results

As shown in Fig. 1b, total *mecA* mRNA in CoNS with multiple SCC*mec* elements exhibited constant and stable expression during the 10 h experiment. However, total *mecA* mRNA in CoNS with single SCC*mec* element was transcribed unsteadily during this experimental course (Fig. 1a). To further assay *mecA* mRNA stability, *mecA* mRNA half-life was analysed in randomly selected *S. epidermidis* H67 and H30. As demonstrated in Fig. 1c, the *mecA* mRNA half-life was approximately 40–50 min in *S. epidermidis* H30. In contrast, *mecA* mRNA was very unstable in *S. epidermidis* H67, in which the *mecA* mRNA half-time probably was no more than 10 min.

**Fig. 2:**
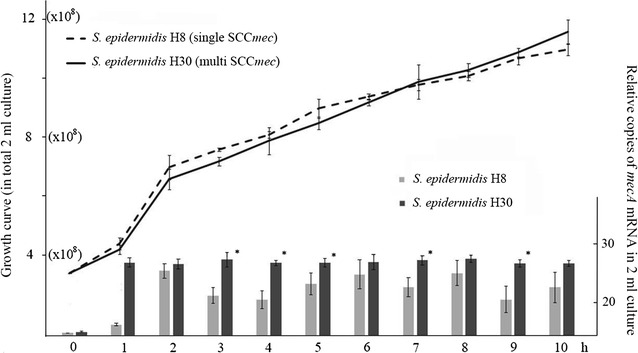
Growth curve indicated total cells in a 2-ml culture of *S. epidermidis* H8, H30 treated with oxacillin at each time point for 10 h (*upper left*). The corresponding relative total *mecA* mRNA in 2-ml sample treated with oxacillin at each time point was measured by quantitative RT real-time PCR (*lower right*). Data were presented as the relative copies of *mecA* mRNA levels compared with that of *S. epidermidis* H8 (0 h).* Each bar* represents the mean ± SD of at least three independent experiments. *P < 0.05 between two strains at each time point

Quantitative RT-PCR analyses of randomly selected isolates further confirmed that the total *mecA* mRNA in *S. epidermidis* H30 demonstrated continuously sustainable expression during the 10 h experiment. In contrast, the total *mecA* mRNA in *S. epidermidis* H8 showed very unstable transcription during this experimental course. Furthermore, the number of *mecA* mRNA relative copies of *S. epidermidis* H8 at many time points was significantly less than that of *S. epidermidis* H30 (lower right panel in Fig. 2). However, no significant differences in growth curves were observed between these two isolates (upper left panel in Fig. 2). Moreover, qRT-PCR of *mecA* mRNA transcription and growth curve assays in other *S. epidermidis* isolates and CoNS species also demonstrated the same phenomenon (Additional file 2: Figure S1).

### CoNS with multiple SCC*mec* elements demonstrated better bacterial cell wall integrity than those carrying a single element

**Fig. 3:**
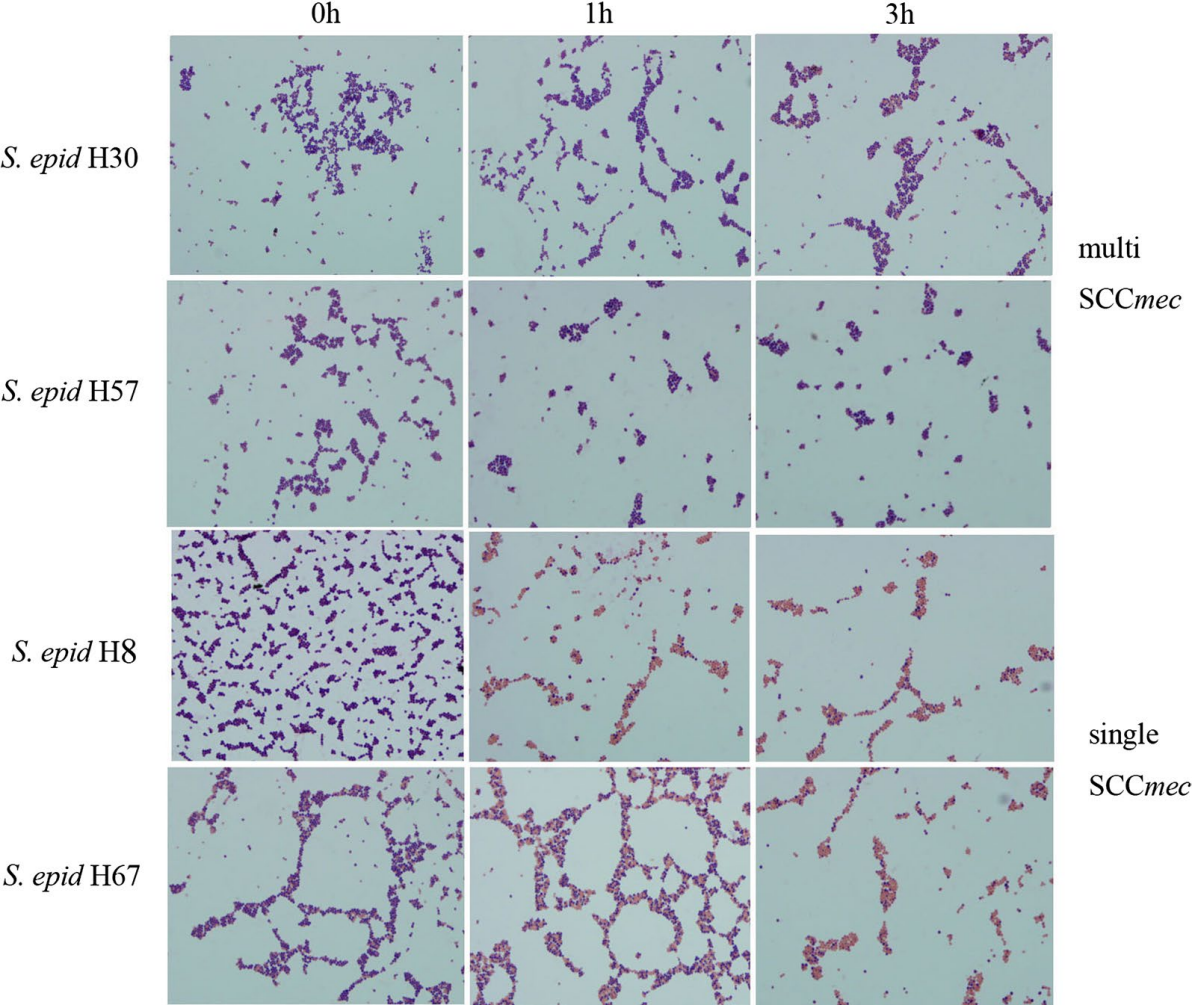
Bacterial cell wall integrity under oxacillin treatment (8 μg/ml). Samples were collected for Gram staining at 0, 1, and 3 h to visualize the bacterial cell wall under a microscope. The proportion of bacteria cells demonstrated as Gram-negative among every 100 total cells, was manually calculated according to three microscope fields for each sample. The results are shown as the mean value of three microscope fields. Data are shown as the images of three independent experiments with similar results

Randomly selected *S. epidermidis* H30, H57 (with multiple SCC*mec* elements) and *S. epidermidis* H8, H67 (with single SCC*mec*) were recruited for analyses of bacterial cell wall integrity. As shown in Fig. 3, most cells of *S. epidermidis* H57, H30 still demonstrated Gram-positivity after 3 h treatment of oxacillin (91.6 ± 1.3, 82.6 ± 2.8%, respectively). In contrast, a smaller number of cells of *S. epidermidis* H8, H67 demonstrated Gram-positivity at 3 h under oxacillin treatment (12.3 ± 1.7, 14.2 ± 2.6%, respectively). The differences between *S. epidermidis* H30, H57 and *S. epidermidis* H8, H67 were significant (P < 0.001).

### Phylogenetic association of *ccr* among CoNS and *S. aureus*

**Fig. 4:**
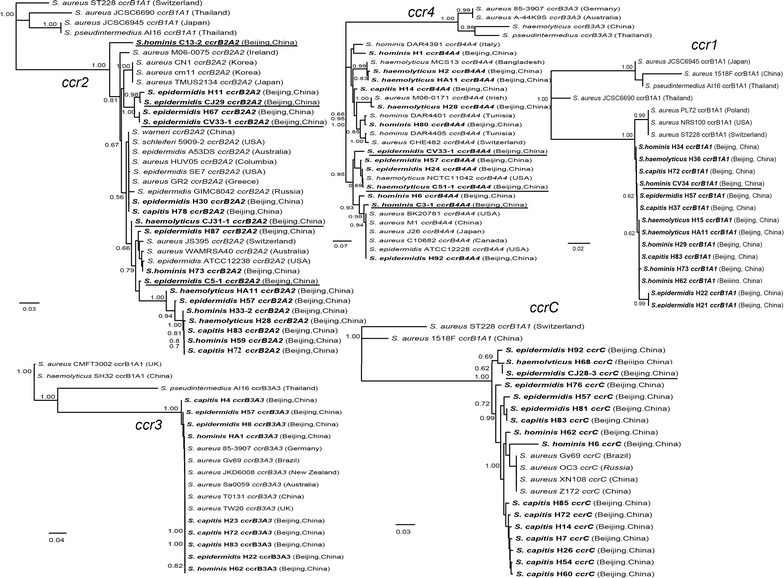
Neighbour-joining tree based on *ccr* sequences determined in this study and those downloaded from GenBank. Those with *bold italic* font represent *ccr* sequences recovered in this study and those *underlined* represent isolates from the community

A total of 70 *ccr* complex segments were sequenced successfully in this study, including 60 clinical isolates and 10 community isolates (Additional file 1: Tables S3, S4). No new *ccr* allotypes or alleles were identified. The *ccrAB* alleles were assigned based on a BLAST search with sequences in GenBank, resulting in 13 *ccrA1B1*, 13 *ccrA2B2*, 9 *ccrA3B3*, 10 *ccrA4B4* and 15 *ccrC* from clinical CoNS and 1 *ccrA1B1*, 5 *ccrA2B2*, 3 *ccrA4B4*, and 1 *ccrC* from community CoNS. As shown in Fig. 4, all species of CoNS recovered from patients contained various types of *ccr* complexes except *ccrA3B3*, which had not been discovered from *S. haemolyticus* in this study. For community isolates, *ccrA3B3* had not been recovered in this study. In each phylogenetic tree, *ccr* segments recovered from clinical CoNS were grouped with those from community isolates in this study and those from around the world. No specific cluster formed for either the clinical isolates or community isolates.

Importantly, *ccrA3B3* segments in clinical CoNS demonstrated high similarity (nearly 100%) with those in *S. aureus* worldwide. In contrast, *ccrA1B1* segments from both the clinic and community were grouped together but separated from those found in *S. aureus*. Interestingly, *ccrA2B2*, *ccrA4B4* and *ccrC* showed an intermediate state, i.e., some clustered with those in *S. aureus* and others had separated from them.

## Discussion

In general, the existence of different types of SCC*mec* in MR-CoNS is dependent on the host species and geographical locations [11]. In this study, SCC*mec* types, II, III, and V were prevalent in MR-CoNS in Beijing. Another study performed from southwest China demonstrated that SCC*mec* types III, IV and V were dominant in MR-CoNS [14]. Moreover, specific SCC*mec* types (in some instances, specific *ccr* or *mec* complex genes) were found particularly in specific CoNS species. Consistent with previous reports, type IV SCC*mec* was preferentially associated with *S. epidermidis* in our study. However, most type V SCC*mec* had been recovered in *S. capitis* in this study, unlike previous study which demonstrated type V dominates in *S. haemolyticus* [10–12]. Although in this study class C *mec* was dominantly associated with *S. haemolyticus* as previous reports [21, 22], *most S. haemolyticus* isolates (clinic or community) carried new SCC*mec* type. We also did not find type VII SCC*mec* in CoNS in this study as other researchers; however, one isolate of type IX was disclosed in *S. haemolyticus* in clinic, which had not been previously confirmed in CoNS [12, 23]. A recent study also reported SCC*mec* type IX in CoNS in the community [24]. Significantly, most CoNS strains recovered in this study (clinic and community) consisted of untypable SCC*mec* elements as many other reports [14–16, 23, 25]. Importantly, the differences between clinical CoNS and community isolates might result from sampling bias as most CoNS recovered from the community were *S. epidermidis*.

Intriguingly, various combinations of *ccr* types were revealed in a single CoNS strain, including clinical and community isolates in this study. Multiple copies of *ccr* complex have been reported in *S. aureus*, *S. epidermidis* and other CoNS. However, most are combinations of *ccrAB* and *ccrC* [6, 25]. To the best of our knowledge, this is the first report of a heterogeneous combination of *ccr* complex in a single CoNS strain, particularly all five types of *ccr* complexes existing in a single clinical isolate. Thus, it was not surprising that these CoNS strains contained multiple SCC*mec* elements. Multiple SCC*mec* elements have been reported in clinical MR-CoNS and the incidences were as high as that observed in our finding [6, 14]. Although it was likely that the two SCC*mec* elements actually constituted a composite rather than two independent units, multiple copies of *mecA* existed in one single CoNS strain both in the clinic and community as revealed in our study. Intriguingly, whether the existence of multiple SCC*mec* in community isolates was attributed to spill over from the hospital or to antibiotic abuse in the community requires further investigation.

It is well-known that acquisition of antibiotic resistance in bacterial cells is often accompanied by fitness cost in the absence of antibiotics, most of which demonstrated slower growth rates and finally resulted in the dilution of antibiotic resistant genes [26]. However, we did not identify any significant differences in the growth rates between CoNS strains with multiple SCC*mec* and those with a single isolate with or without oxacillin, and no correlation of multiple SCCmec with MIC in response to oxacillin was disclosed (data not shown). We speculated that MIC in response to oxacillin might also be correlated with other antibiotic genes [27]. Interestingly, we demonstrated that multiple SCC*mec* elements in CoNS strains ensure stable and continuous transcription of antibiotic-resistant genes, i.e., *mecA* gene, whose transcript, PBP2a, was capable of maintaining cell wall integrity [28]. Further analyses by Gram staining demonstrated that the cell wall in CoNS with multiple SCC*mec* demonstrated much stronger resistance and better integrity under oxacillin treatment than those CoNS with single SCC*mec* element. Interestingly, although the bacteria cell number dramatically increased, total *mecA* mRNA levels sustained constantly from 1 h to 10 h incubation with oxacillin. We suspected that the amount of *mecA* mRNA at the 1 h time point was sufficient to resist the antibiotics added into the culture.

Finally, phylogenetic analyses of *ccr* indicated potential horizontal gene transfer among different CoNS species of clinic and community isolates, even among CoNS and *S. aureus*. In particular, nearly 100% similarity of all ccr*A3B3* might result from a recent gene transfer among different CoNS species and *S. aureus*. As observed in previous studies [16], we also detected the *ccr* complex in *mec* negative strains. Specifically, some community CoNS strains had multiple *ccr* but lacked the *mec* gene. Their potential to acquire *mec* genes with these *ccr* complexes requires further attention. However, the limitation of our work is that we could not determine which *ccr* complex was linked to the specific SCC*mec*, particularly for those that had two types of *mecA* classes (multiple SCC*mec*). The presence of untypable and multiple SCC*mec* elements represent great challenges for SCC*mec* typing in MR-CoNS [11, 14, 15, 23]. Whole genome sequencing of more MR-CoNS would be helpful to construct a new typing method through understanding the relative position and precise composition of multiple SCC*mec* and to further elucidate the role of *ccr* complexes in spreading SCC*mec* elements among CoNS and *S. aureus*.

## Conclusions

Overall, CoNS recovered in Beijing carried extremely diverse SCC*mec* elements including multiple SCC*mec* elements, which demonstrated superior cell wall integrity. Our data revealed potential horizontal gene transfer among different CoNS species of clinic and community isolates, even among CoNS and *S. aureus.*

## Additional files

**Additional file 1: Table S1.** Primers used for *mec* gene detection and SCC*mec* Typing. **Table S2.** The *ccr* gene sequences obtained from GenBank for phylogenetic analyses. **Table S3.** The *ccr* gene sequences obtained in this study for phylogenetic analyses. **Table S4.** Origins of the CoNS strains whose *ccr* segments were applied to phylogenetic analyses.

**Additional file 2: Figure S1.** Growth curve indicating the total cells in a 2-ml culture of CoNS treated with oxacillin at each time point for 10 h (upper left in panel A, B, and C). The corresponding relative total *mecA* mRNA in the 2-ml sample treated with oxacillin at each time point was measured by quantitative RT real-time PCR (lower right in each panel). Data are presented as the relative copies of *mecA* mRNA levels compared with that of *S. epidermidis* H8 (0 h). Each bar represents the mean±SD of at least three independent experiments. *P<0.05 between two strains at each time point.

## Abbreviations

CoNS: coagulase-negative staphylococci
SCC*mec*: staphylococcal cassette chromosome *mec*
*ccr*: cassette chromosome recombinase
MIC: minimal inhibitory concentrations
