# Supplementary material for: Extreme diversity and multiple SCCmec elements in coagulase-negative Staphylococcus found in the Clinic and Community in Beijing, China
Source: Ann Clin Microbiol Antimicrob. 2017 Aug 22;16:57. doi: 10.1186/s12941-017-0231-z (PMC5568392; doi:10.1186/s12941-017-0231-z)
Supplement: Supplementary file 1 — Additional file 1: Table S1. Primers used for mec gene detection and SCCmec Typing. Table S2. The ccr gene sequences obtained from GenBank for phylogenetic analyses. Table S3. The ccr gene sequences obtained in this study for phylogenetic analyses. Table S4. Origins of the CoNS strains whose ccr segments were applied to phylogenetic analyses. [file 12941_2017_231_MOESM1_ESM.docx]

**Table S1**. Primers used for *mec* gene detection and SCC*mec* Typing

| Gene | Primer names | Primer sequence | Length | Reference |
| --- | --- | --- | --- | --- |
| *mecA* | F: mecA-1501F | GCTCAAATTTCAAACAAAAATTTAGATAATG | 98 bp | 19 |
|  | R:mecA-1598R | TGAAAGGATCTGTACTGGGTTAATCAGT |  |  |
| *mecA* | F: met1 | GGG ATC ATA GCG TCA TTA TTC | 527 bp | 16 |
|  | R: met2 | AAC GAT TGT GAC ACG ATA GCC |  |  |
| *mecA* | F: mecAF | ATGAGATTAGGCATCGTTCC | 554 bp | 18 |
|  | R: mecAR | TGGATGACAGTACCTGAGCC |  |  |
| *mecC* | F: mecCF | TCACCAGGTTCAAC[Y]CAAAA | 356 bp | 5 |
|  | R: mecCR | CCTGAATC[W]GCTAATAATATTTC |  |  |
| class A *mec* | F: mA7 | ATATACCAAACCCGACAACTACA | 1963 bp | 6 |
|  | R: mI6 | CATAACTTCCCATTCTGCAGATG |  |  |
| class B *mec* | F: mA7 | See as above | 2827 bp | 6 |
|  | R: IS7 | ATGCTTAATGATAGCATCCGAATG |  |  |
| class C *mec* | F: mA7 | See above | 804 bp | 6 |
|  | R: IS2 | TGAGGTTATTCAGATATTTCGATGT |  |  |
| class C1 *mec* | F: mA7 | See above | 804 bp | 11 |
|  | R: IS2L | GAACCGCAGGTCTCTTCAGATC |  |  |
| around IS431 | IS431-F2 | GGTCTACCGTTGGGTTCAAG |  | 14 |
|  | IS431-R1 | CGTCTCATCAATACGCCATTT |  |  |
|  | mecA-R2 | TCGGACGTTCAGTCATTTCT |  |  |
| *ccrA1B1* | F: A1 | AACCTATATCATCAATCAGTACGT | 695 bp | 6 |
|  | R: BC | ATTGCCTTGATAATAGCCITCT |  |  |
| *ccrA2B2* | F:A2 | TAAAGGCATCAATGCACAAACACT | 937 bp | 6 |
|  | R: BC | See above |  |  |
| *ccrA3B3* | F: A3 | AGCTCAAAAGCAAGCAATAGAAT | 1791 bp | 6 |
|  | R: BC | See above |  |  |
| *ccrA4B4* | F: a4U | GCGACGAATCAAATGTCCTTACTG | 1304 bp | 11 |
|  | R: b4U | ATCGCTCCAGTGTCTATACTTCGC |  |  |
| *ccrC* | F: rf | CGTCTATTACAAGATGTTAAGGATAAT | 607 bp | 11 |
|  | R: ccrCU1 | TTACCTTTGACCAATATCACATC |  |  |

**Table S2**. The *ccr* gene sequences obtained from GenBank for phylogenetic analyses.

| Species | Strain | *ccr* type | GenBank no. | Location |
| --- | --- | --- | --- | --- |
| *S. aureus* | 1518F | *ccrB1A1* | KP777553.1 | China |
|  | PL72 | *ccrB1A1* | AB433542.1 | Poland |
|  | NRS100 | *ccrB1A1* | CP007539.1 | USA |
|  | ST228 | *ccrB1A1* | HE579073.1 | Switzerland |
|  | CMFT3002 | *ccrB1A1* | HF569102.1 | UK |
|  | JCSC6690 | *ccrB1A1* | AB705452.1 | Thailand |
|  | JCSC6945 | *ccrB1A1* | AB505630.1 | Japan |
| *S. pseudintermedius* | AI16 | *ccrB1A1* | LN864705.1 | Thailand |
| *S. haemolyticus* | SH32 | *ccrB1A1* | KF006347.1 | China |
| *S. aureus* | M06/0075 | *ccrB2A2* | AM983545.1 | Ireland |
|  | CN1 | *ccrB2A2* | CP003979.1 | Korea |
|  | cm11 | *ccrB2A2* | EU437549.2 | Korea |
|  | TMUS2134 | *ccrB2A2* | AP014653.1 | Japan |
|  | HUV05 | *ccrB2A2* | CP007676.1 | Columbia |
|  | GR2 | *ccrB2A2* | CP010402.1 | Greece |
|  | JS395 | *ccrB2A2* | CP012756.1 | Switzerland |
|  | WAMRSA40 | *ccrB2A2* | JQ746621.1 | Australia |
| *S. warneri* | DY39 | *ccrB2A2* | KU170612.1 | China |
| *S. schleiferi* | 5909-2 | *ccrB2A2* | CP009676.1 | USA |
| *S. epidermidis* | A-53DS | *ccrB2A2* | DQ196433.1 | Australia |
|  | SE7 | *ccrB2A2* | DQ514333.1 | USA |
|  | GIMC8042 | *ccrB2A2* | KF056793.1 | Russia |
|  | ATCC12238 | *ccrB2A2* | BK001539.1 | USA |
| *S. aureus* | 85-3907 | *ccrB3A3* | AB047088.2 | Germany |
|  | A-44K9S | *ccrB3A3* | DQ196432.1 | Australia |
|  | Gv69 | *ccrB3A3* | CP009681.1 | Brazil |
|  | JKD6008 | *ccrB3A3* | CP002120.1 | New Zealand |
|  | Sa0059 | *ccrB3A3* | JQ412578.1 | Australia |
|  | T0131 | *ccrB3A3* | CP002643.1 | China |
|  | TW20 | *ccrB3A3* | FN433596.1 | UK |
| *S. pseudintermedius* | AI16 | *ccrB3A3* | LN864705.1 | Thailand |
| *S. hominis* | DAR4391 | *ccrB4A4* | JQ836538.1 | Italy |
| *S. haemolyticus* | MCS13 | *ccrB4A4* | AB587081.1 | Bangladesh |
| *S. aureus* | M06-0171 | *ccrB4A4* | HE980450.1 | Irish |
| *S. hominis* | DAR4401 | *ccrB4A4* | JQ836541.1 | Tunisia |
|  | DAR4405 | *ccrB4A4* | JQ836543.1 | Tunisia |
| *S. aureus* | CHE482 | *ccrB4A4* | EF126185.1 | Switzerland |

(to be continued)

| Species | Strain | *ccr* type | GenBank no. | Location |
| --- | --- | --- | --- | --- |
| *S. haemolyticus* | NCTC11042 | *ccrB4A4* | AB505631.1 | USA |
| *S. aureus* | BK20781 | *ccrB4A4* | FJ670542.1 | USA |
| *S. aureus* | M1 | *ccrB4A4* | NZ_LIDQ00000000.1 | China |
| *S. aureus* | J266 | *ccrB4A4* | AB774374.1 | Japan |
| *S. aureus* | C10682 | *ccrB4A4* | FJ390057.1 | Canada |
| *S. epidermidis* | ATCC12228 | *ccrB4A4* | BK001539.1 | USA |
| *S. aureus* | Gv69 | *ccrC* | CP009681.1 | Brazil |
| *S. aureus* | OC3 | *ccrC* | NZ_BBKC01000008.1 | Russia |
| *S. aureus* | XN108 | *ccrC* | CP007447.1 | China |
| *S. aureus* | Z172 | *ccrC* | NC_022604.1 | China |

| Species | Strain | GenBank no. | | | | |
| --- | --- | --- | --- | --- | --- | --- |
|  |  | *ccrA1B1* | *ccrA2B2* | *ccrA3B3* | *ccrA4B4* | *ccrC* |
| *S.epidermidis* | H21 | KX697924 | — | — | — | — |
|  | H8 | — | — | KX697919 | — | — |
|  | H22 | KX697925 | — | KX697926 | — | — |
|  | H24 | — | — | — | KX697928 | — |
|  | H30 | — | KX697863 | — | — | — |
|  | H57 | KX697869 | KX697870 | KX697871 | KX697872 | KX697873 |
|  | H76 | — | — | — | — | KX697887 |
|  | H87 | — | KX697896 | — | — | — |
|  | H81 | — | — | — | — | KX697890 |
|  | H92 | — | — | — | KX697897 | KX697898 |
|  | H11 | — | KX697920 | — | — | — |
|  | H67 | — | KX697879 | — | — | — |
|  | C5-1 | — | KX697904 | — | — | — |
|  | CJ28-3 | — | — | — | — | KX697907 |
|  | CJ29 | — | KX697908 | — | — | — |
|  | CV33-1 | — | KX697910 |  | KX697911 | — |
| *S.capitis* | H4 | — | — | KX697915 | — | — |
|  | H7 | — | — | — | — | KX697918 |
|  | H14 | — | — | — | KX697921 | KX697922 |
|  | H23 | — | — | KX697927 | — | — |
|  | H26 | — | — | — | — | KX697929 |
|  | H37 | KX697867 | — | — | — | — |
|  | H54 | — | — | — | — | KX697868 |
|  | H60 | — | — | — | — | KX697875 |
|  | H72 | KX697881 | KX697882 | KX697883 | — | KX697884 |
|  | H78 | — | KX697888 | — | — | — |
|  | H83 | KX697891 | KX697892 | KX697893 | — | KX697894 |
|  | H85 | — | — | — | — | KX697895 |
| *S.hominis* | HA1 | — | — | KX697899 | — |  |
|  | H1 | — | — | — | KX697913 |  |
|  | H6 | — | — | — | KX697916 | KX697917 |
|  | H29 | KX697862 | — | — | — | — |
|  | H33-2 | — | KX697864 | — | — | — |
|  | H34 | KX697865 | — | — | — | — |
|  | H59 | — | KX697874 | — | — | — |

**Table S3**. The *ccr* gene sequences obtained in this study for phylogenetic analyses.

(to be continued)

| Species | Strain | GenBank no. | | | | |
| --- | --- | --- | --- | --- | --- | --- |
|  |  | *ccrA1B1* | *ccrA2B2* | *ccrA3B3* | *ccrA4B4* | *ccrC* |
| *S.hominis* | H62 | KX697876 | — | KX697877 | — | KX697878 |
|  | H73 | KX697885 | KX697886 | — | — | — |
|  | H80 | — | — | — | KX697889 | — |
|  | C3-1 | — | — | — | KX697903 | — |
|  | C13-2 | — | KX697905 | — | — | — |
|  | CV34 | KX697912 | — | — | — | — |
| *S.haemolyticus* | H2 | — | — | — | KX697914 | — |
|  | H15 | KX697923 | — | — | — | — |
|  | H28 | — | KX697930 | — | KX697931 | — |
|  | H36 | KX697866 | — | — | — | — |
|  | H68 | — | — | — | — | KX697880 |
|  | HA11 | KX697900 | KX697901 | — | KX697902 | — |
|  | CJ31-1 | — | KX697909 | — | — | — |
|  | C51-1 | — | — | — | KX697906 | — |

—, not available

**Table S4**. Origins of the CoNS strains whose *ccr* segments were applied to phylogenetic analyses

| Species | Strain | Source | Species | Strain | Source |
| --- | --- | --- | --- | --- | --- |
| *S.epidermidis* | H21 | (Hb) ICU2 | *S.hominis* | HA1 | (Ha)hematology department |
|  | H22 | (Hb) ICU1 |  | H1 | (Hb) ICU2 |
|  | H24 | (Ha)hyperbaric oxygen chamber |  | H6 | (Hb) ICU1 |
|  | H30 | (Hb)hyperbaric oxygen chamber |  | H29 | (Hb)hematology department |
|  | H57 | (Hb)Emergency ICU |  | H33-2 | (Hb)hematology department |
|  | H76 | (Hb) ICU1 |  | H34 | (Ha) ICU |
|  | H87 | (Hb)Orthopedics department |  | H59 | (Hb)hematology department |
|  | H81 | (Hb) ICU2 |  | H62 | (Hb) ICU2 |
|  | H92 | (Hb) ICU1 |  | H73 | (Hb) ICU2 |
|  | H11 | (Ha) ICU |  | H80 | (Hb)hyperbaric oxygen chamber |
|  | H67 | (Hb) ICU1 |  | C3-1 | (C) Changping |
|  | C5-1 | (C) Changping |  | C13-2 | (C) Changping |
|  | CJ28-3 | (C) Changping |  | CV34 | (C) Changping |
|  | CJ29 | (C) Changping | *S.haemolyticus* | H2 | (Hb)nephrology department |
|  | CV33-1 | (C) Changping |  | H15 | (Hb)preventive medicine |
| *S.capitis* | H4 | (Hb) ICU2 |  | H28 | (Ha) ICU |
|  | H7 | (Hb)hyperbaric oxygen chamber |  | H36 | (Hb) ICU1 |
|  | H14 | (Hb) ICU1 |  | H68 | (Hb)Emergency ICU |
|  | H23 | (Hb)hyperbaric oxygen chamber |  | HA11 | (Ha)hematology department |
|  | H26 | (Hb) ICU1 |  | CJ31-1 | (C) Changping |
|  | H37 | (Hb)Emergency ICU |  | C51-1 | (C) Changping |
|  | H54 | (Hb) ICU2 |  |  |  |
|  | H60 | (Ha) ICU |  |  |  |
|  | H72 | (Hb)Emergency ICU |  |  |  |
|  | H78 | (Hb) ICU2 |  |  |  |
|  | H83 | (Ha)preventive medicine |  |  |  |
|  | H85 | (Hb) ICU1 |  |  |  |

Note: (Ha), hospital a; (Hb), hospital b; (C), community
