# Supplementary figures and images for: Extreme diversity and multiple SCCmec elements in coagulase-negative Staphylococcus found in the Clinic and Community in Beijing, China
Source: Ann Clin Microbiol Antimicrob. 2017 Aug 22;16:57. doi: 10.1186/s12941-017-0231-z (PMC5568392; doi:10.1186/s12941-017-0231-z)

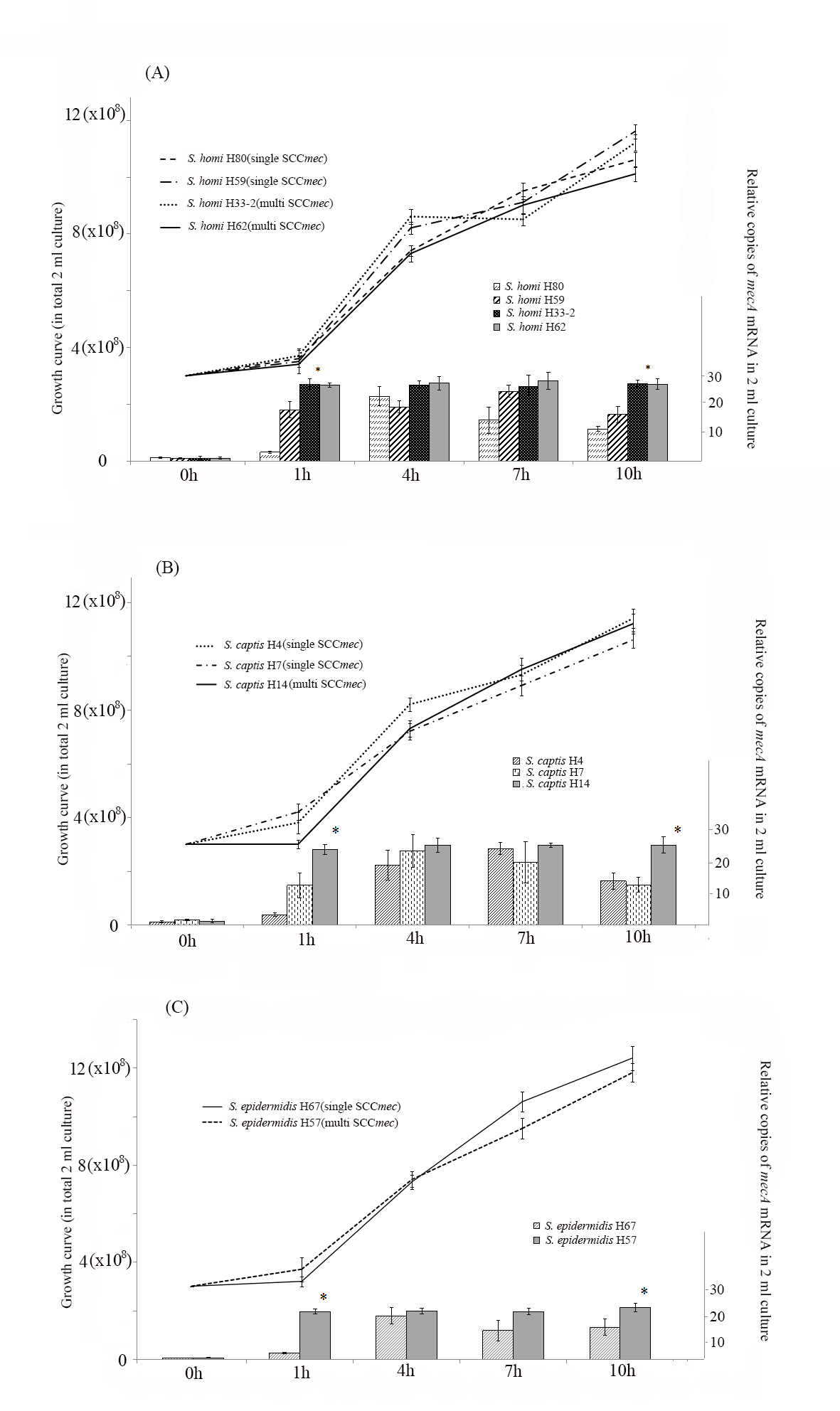

Supplement: Supplementary file 2 — Additional file 2: Figure S1. Growth curve indicating the total cells in a 2-ml culture of CoNS treated with oxacillin at each time point for 10 h (upper left in panel A, B, and C). The corresponding relative total mecA mRNA in the 2-ml sample treated with oxacillin at each time point was measured by quantitative RT real-time PCR (lower right in each panel). Data are presented as the relative copies of mecA mRNA levels compared with that of S. epidermidis H8 (0 h). Each bar represents the mean±SD of at least three independent experiments. *P<0.05 between two strains at each time point. [file 12941_2017_231_MOESM2_ESM.tif]
